# Chitosan/PVA Nanofibers as Potential Material for the Development of Soft Actuators

**DOI:** 10.3390/polym15092037

**Published:** 2023-04-25

**Authors:** Rigel Antonio Olvera Bernal, Roman Olegovich Olekhnovich, Mayya Valerievna Uspenskaya

**Affiliations:** Chemical Engineering Centre, ITMO University, Kronverkskiy Prospekt, 49A, St. Petersburg 197101, Russia

**Keywords:** chitosan, PVA, nanofibers, EAPs, electrospinning, deconvolution

## Abstract

Chitosan/PVA nanofibrous electroresponsive soft actuators were successfully obtained using an electrospinning process, which showed fast speed displacement under an acidic environment. Chitosan/PVA nanofibers were prepared and characterized, and their electroactive response was tested. Chitosan/PVA nanofibers were electrospun from a chitosan/PVA solution at different chitosan contents (2.5, 3, 3.5, and 4 wt.%). Nanofibers samples were characterized using Fourier transform infrared analyses, thermogravimetric analysis (TGA), differential scanning calorimetry (DSC), optical microscopy, and tensile test. The electroactive behavior of the nanofiber hydrogels was tested under different HCl pH (2–6) under a constant voltage (10 V). The electroactive response test showed a dependence between the nanofiber’s chitosan content and pH with the bending speed displacement, reaching a maximum speed displacement of 1.86 mm^−1^ in a pH 3 sample with a chitosan content of 4 wt.%. The results of the electroactive response were further supported by the determination of the proportion of free amine groups, though deconvoluting the FTIR spectra in the range of 3000–3700 cm^−1^. Deconvolution results showed that the proportion of free amine increased as the chitosan content was higher, being 3.6% and 4.59% for nanofibers with chitosan content of 2.5 and 4 wt.%, respectively.

## 1. Introduction

Regarding the evolution of robotics, it is possible to observe that the aim is to develop a system capable of fully imitating a biological system. This trend is referred to as Biomimetics, which represents the study and imitation of nature’s methods, designs, and processes [1]. One of the biggest challenges faced in the field of robotics is trying to mimic the actuation systems that endow animals with locomotion. New materials have been developed addressing this situation; these materials are often referred to as soft actuators or artificial muscles [2], which can be defined as materials capable of changing their shape or size in response to physical stimuli such as light, humidity, pH, electricity, and so forth [3]. To date, many technologies have been developed for their use as soft actuators driven by various types of external stimuli, such as magnetically responsive actuators [4,5], shape memory polymers (SMPs) [6,7], and pneumatic actuators [8,9].

Among the many materials that can serve as soft actuators, electroactive polymers have gained the attention of scientists and engineers due to characteristics such as large deformation, flexibility, low density, tuneability, and ease of manufacture. Moreover, they exhibit a similar actuation behavior to biological muscles. Electroactive polymers (EAPs) can be classified into two major groups, depending on their actuation mechanism. In the first group are electronic EAPs (which are driven by electric field or coulomb forces), including ferroelectric polymers, dielectric elastomers, and liquid crystal elastomers. The second group is composed of ionic EAPs (which change shape by mobility or diffusion of ions and their conjugated substances), including ionic polymer gels, ionic polymer–metal composites (IPMC), conductive polymers, and others [10,11,12]. Among EAPs, dielectric elastomer actuators (DEAs) and liquid crystal elastomers have been widely studied for the development of soft actuators due to their flexibility, high energy density, high strain, and good electromechanical actuation performance. However, both DEAs and liquid crystal elastomers have disadvantages, such as high activation voltages (in kV), which can lead to dielectric breakdown [13,14]. In contrast, hydrogel materials have been extensively studied due to their ability to respond to various external stimuli such as light, magnetic fields, pH, and electric fields [15].

Electroactive hydrogels are a type of electroresponsive material belonging to the group of ionic EAPs. More specifically, these materials fall into the subcategory of ion-exchange polymer–metal composites (IPMCs), whose actuation is governed by the transport or migration of ions inside their three-dimensional polymeric networks [16]. Electroactive hydrogel exhibits large deformation in response to an electric field, fast actuation, and biomimetic materials properties [17]. Hydrogel-based nanofibers are a relatively new class of nanomaterials where hydrogels are structured in nanofibrous form. Hydrogel nanofiber combines the desirable properties of both hydrogel and nanofiber, such as flexibility, soft consistency, elasticity, and others [18]. Although hydrogels are great candidates for the development of stimuli-responsive materials [19], nanofibers stand out as electroresponsive materials due to their morphology. Due to the fact that the rate of actuation is inversely proportional to the size of the hydrogel, reducing the size will promote a shorter response time [20]. Because nanofibers are one-dimensional materials with a high surface-to-volume ratio, the surface area of each fiber is greater, increasing the ionization of functional groups when interacting with the medium. In addition, the high porosity of the nanofiber mat optimizes the diffusion of free ions migrating from the inside to the outside of the material and vice versa, increasing the deformation of the material and improving the mechanical strength in relation to hydrogels.

In recent decades, biopolymers have been studied as potential electroactive materials [21]. Natural polymers such as chitosan, cellulose, starch, and gelatin, among others, exhibit good electromechanical properties due to their polar groups, which are vulnerable to polarization, such as amino, hydroxyl, and carboxyl [22]. Chitosan is a linear polysaccharide composed of randomly distributed deacetylated units (β-(1,4)-D-glucosamine) and acetylated units (N-acetyl-D-glucosamine). Chitosan has amino groups and hydroxyl groups on its backbone, which gives chitosan a polycationic property [23,24]. Due to its polycationic characteristic, chitosan has been studied for the development of electroresponsive materials. Emine et al. [25] reported a chitosan–PDAD (poly (diallyldimethylammonium chloride)) base actuator. Wang et al. [26], with the use of chitosan and cellulose, developed an electroactive paper (EAPap) actuator. Jinzhu Li et al. [27], using carbon nanotubes, electrodes, and chitosan, developed an actuator with a superfast response (~19 ms) under low voltages (>8 V).

Polyvinyl alcohol (PVA) is a synthetic, linear, semicrystalline polymer that is composed of a carbon chain as the backbone and a hydroxyl group as a functional group. PVA exhibits many important features; for example, it is readily available, water-soluble, has excellent film-forming ability, and is thermostable, along with others [28]. Furthermore, it can also aid in improving fiber spinning by reducing repulsive forces within charged polymer solutions [29,30].

Electrospinning is a process in which a high voltage is applied to a polymeric solution injected through a capillary or needle. An electrostatic charge accumulates at the tip of the droplet; therefore, charge repulsion overcomes the surface tension of the droplet generating a cone-like jet (commonly known as a Taylor cone) and is collected on a grounded electrode plate [31,32]. During the electrospinning process, factors such as technical parameters (flow rate, voltage, and needle collector distance), solution parameters (conductivity, viscosity, polymer concentration), and environmental parameters have a direct influence on the morphology of the electrospun fibers [33]. This method allows the formation of a hierarchical architecture that could help mimic the fibrous morphology of biological muscles when developing nanofiber-based electroactive gels.

Many works have been devoted to the study of fibrous hydrogels as soft actuators. Miranda et al. [34] reported a hybrid nanofiber hydrogel based on poly (acrylamide and acrylic acid) mixed with aniline; the electroactive response was assessed in an electrochemical cell. The hybrid material exhibited a high electrical conductivity, reaching values near 0.01 S cm^−1^, as well as a fast time response (2.5 mm s^−1^) and reversible displacement at low electrical potential (1 V) in 1 MΩ cm distilled water. Ismail et al. [35] proposed a novel approach for the fabrication of flexible hydrogel nanofiber actuators based on PVA/PANI. The hybrid actuator had a maximum linear actuation strain of 1.8% in 1 M methane sulfonic acid solution and showed a stable actuation in extended electrochemical cycles reaching up to 250 cycles.

Nevertheless, the number of works reporting the use of biopolymers as a base material for the fabrication of nanofiber hydrogels as soft actuators are very limited. In this work, the use of chitosan at different concentrations as a base material for the production of nanofibers hydrogel as a potential material for the development of soft actuators is reported. The electrospinning method was utilized for the formation of chitosan/PVA nanofiber mats. The electroactive response of the material was tested at different pH of HCl solution at a constant electrical potential, and the displacement speed (mm s^−1^) was measured for each sample.

## 2. Materials and Methods

### 2.1. Materials

Chitosan (MW 260 kDa) and PVA (MW 74 kDa) were used as received from the Russian Federation limited liability company “Bioprogres”. Acetic acid (99.5%) and distilled water were used as components of the binary solvent system.

### 2.2. Preparation of Polymeric Solutions

Chitosan was dissolved in an Acetic Acid/Distilled Water (80/20) binary solvent system using magnetic stirring at 90 °C to prepare 5, 6, 7, and 8 wt.% solutions. PVA solutions (10 wt.%) were prepared by dissolving PVA powder in distilled water while maintaining a constant temperature of 80 °C. Chitosan and PVA solutions were mixed in a volume ratio of 1:1 in order to prepare solutions with different chitosan concentrations while maintaining a constant PVA concentration. The chitosan and PVA concentrations of the precursor solution were as follows, chitosan 2.5 wt.%/PVA 5 wt.%, chitosan 3 wt.%/PVA 5 wt.%, chitosan 3.5 wt.%/PVA 5 wt.%, and chitosan 4 wt.%/PVA 5 wt.%. The solutions were labeled as Cs-5, Cs-6, Cs-7, and Cs-8, respectively.

### 2.3. Electrospinning Process

PVA–chitosan solutions were electrospun in a NANON-01A system (MECC CO., LTD., Fukuoka, Japan). The electrospinning process was performed at a temperature of 30.0 ± 2 °C and a relative humidity of 35 ± 1%. The technical parameters were kept the same for all solutions. During the process, a drum collector was used to obtain the nanofibers mat. The values used during the electrospun of the fibers were as follows: voltage 30 kV; feed rate 0.2 mL/h; needle-collector distance 150 mm; drum’s rotational speed 500 rpm. For the nanofiber mat formation, a volume of 5 mL of polymeric solution was electrospun for 13 h without any interruptions. The fibers were collected on aluminum foil that was previously placed on the drum collector’s surface.

### 2.4. Morphology and Diameter Determination

For the characterization of the morphology PVA–CS fibers, micrographs were obtained with the use of an optical microscope Olympus STM6 (OLYMPUS Corporation, Tokyo, Japan). The differentially interferential contrasting technique (DIC) was utilized to emphasize the colorfulness and contrast of the obtained fibers. ImageJ (National Institutes of Health, Bethesda, MD, USA) was used for the analysis and measurement of the fibers’ diameter from the obtained microphotographs program. The average diameter of the nanofibers was obtained by measuring the diameter of 200 fibers for each sample.

### 2.5. Infrared Spectroscopy

A Bruker Tensor 37 Fourier transform Infrared (FTIR) spectrometer (Bruker, Germany) was used to obtain the infrared absorption spectra of the samples. The spectra were recorded at a resolution of 2 cm^−1^ in the range of 4000–600 cm^−1^ and a total of 32 scans.

### 2.6. Thermal Properties

Thermogravimetric analysis (TGA) was performed using a TG 209 F1 Libra (Netzsch, Germany) under a nitrogen atmosphere with a flow rate of 40 mL/min. The samples were studied at a range of temperatures from 25 °C to 900 °C with an increasing rate of 10 K/min. Differential scanning calorimetry analysis was performed on a NETZSCH DSC 204F1 Phoenix under a nitrogen atmosphere. Around 4 mg of material (PVA, chitosan, and chitosan/PVA nanofibers) were sealed in aluminum pans. All samples were submitted in two heating cycles; the first heating cycle started at 25 °C reaching 150 °C, then the temperature was dropped down to −30 °C; consequently, the second heating cycle started at −30 °C and ended at 300 °C. The first heating cycle had the purpose of removing water or any solvent residuals from the samples.

### 2.7. Mechanical Properties

The tensile properties were measured using an Instron 5943 tensile testing machine (Instron, Norwood, MA, USA). All samples were tested in accordance with the ISO 527-3 standard at room temperature and a speed test of 10 mm/min. The sample sizes were 120 × 10 mm (length × wide), and the length between grips for each sample was 100 mm, with a thickness of 29, 27, 46, and 29 µm for Cs-5, Cs-6, Cs-7, and Cs-8, respectively. The thickness was measured using a digital micrometer. Sample thickness is the mean value of the thickness measured at three different positions. Four samples of each composition were tested, and averages of Young’s modulus, tensile strength, and elongation at break were evaluated.

### 2.8. Swelling Ratio of Nanofiber Hydrogels

To measure the equilibrium swelling ratio, pre-weighed dry samples were immersed in HCl solutions with different pH values (from 2 to 6) until they swelled to equilibrium. It was confirmed that 24 h equilibration was enough to reach the equilibrium swelling for the samples. After excessive surface water was removed with filter paper, the fully swollen samples were weighed. The equilibrium swelling ratio was calculated from the following equation:

(1) $$Equilibrium\;swelling\;ratio=\left(W_{s}-W_{d}\right)/W_{d}$$

where *W_s_* is the weight of the swollen state of a sample at *equilibrium* and *W_d_* is the weight of the sample at the dry state. The *swelling* experiments were repeated 3 times for each sample.

### 2.9. Electroactive Response

**Figure 1:**
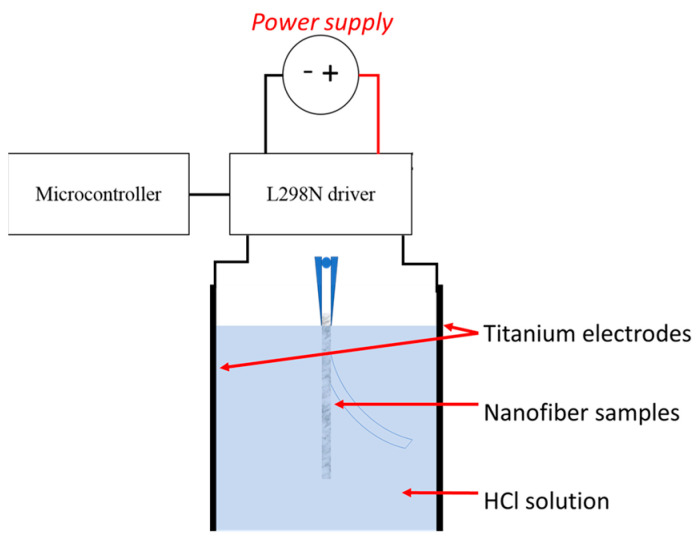
Schematic diagram for testing the electroactive response of the nanofiber samples.

To test the electroactive response of the material, the samples were immersed in HCl solutions at different pHs (from pH 2 to pH 6) at room temperature. For the experiments, all samples used had a dimension of 20 × 3 mm (length × width). All samples were immersed into the solution and left to repose for 30 min prior to each test. The samples were held with the help of a gripper between two titanium electrodes that were placed 15 mm apart from each other. The voltage in all experiments was kept constant at 10 V. The speed displacement was measured for each nanofibers sample at a different pH. Aiming to give higher accuracy in the measurements, a voltage supply of 0–30 V (QJE PS3005 power supply) was connected to an L298N driver. The outputs of the driver were attached to the titanium electrodes, and the polarity in the electrodes was inversed at intervals of 1 s with the use of a microcontroller (Arduino nano) that was connected to the L298N driver; a diagram of the apparatus is shown in Figure 1. The system was left to run by itself for a total of 3 min. The displacement/bending of the nanofibers was recorded with a digital video camera at 30 FPS. Images from different time periods were selected from the resulting video. The selected images were exported to ImageJ software which measured the linear displacement as a function of time. It is important to mention that the displacement speed (mm s^−1^) that is shown was obtained as the main value of different measurements made at different times of the video. For each sample and different pH, 5 measurements were made to obtain the main value.

## 3. Results and Discussion

### 3.1. Morphology and Diameter Distribution of Chitosan/PVA Nanofibers

**Figure 2:**
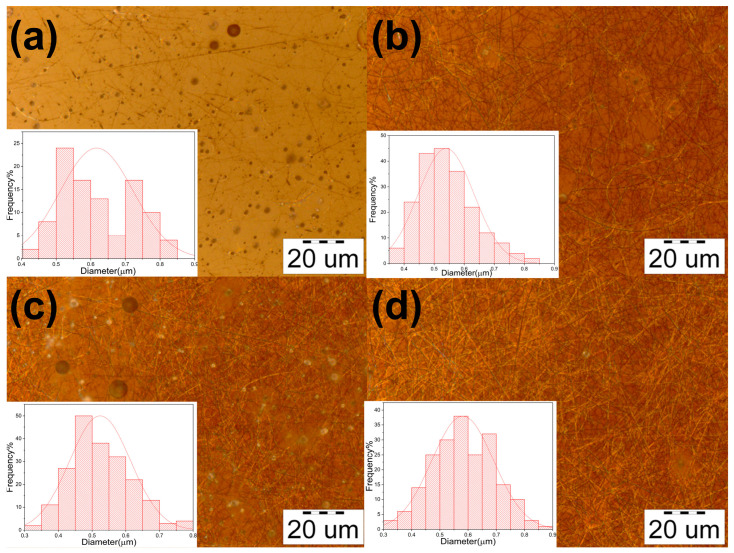
Micrographs of the electrospun fibers. (**a**) chitosan 2.5%/PVA 5%; (**b**) chitosan 3%/PVA 5%; (**c**) chitosan 3.5%/PVA 5%; (**d**) chitosan 4%/PVA 5%.

In Figure 2, nanofibers with 2.5, 3, 3.5, and 4 wt.% of chitosan and 5 wt.% PVA electrospun nanofibers are shown. From the obtained samples, it is possible to observe that the concentration of chitosan has a major influence on the quality of the fibers (Table 1). Chitosan is a cationic polysaccharide with amino groups attached to its backbone. Amino groups react with acetic acid, forming -NH_3_ ions. The protonation of amino groups generates charge repulsions causing the chitosan chains to expand. As a result, the interaction between chitosan and PVA increases, enhancing the fiber formation by maintaining the stability of the ejected jet during electrospinning. It is safe to say that a lower concentration of chitosan diminishes the entanglement and polymer–polymer interaction, thus, causing the ejected jet to lose its fibrous structure, shattering into interconnected sections [36,37,38]. Furthermore, these sections can get split into smaller sections and form spherical shapes; meanwhile, the interconnected parts will form fine filaments, as shown in Figure 2a.

### 3.2. FTIR Spectroscopy

With the purpose of investigating the molecular interaction in the chitosan/PVA nanofibers, the FTIR spectrum of chitosan, PVA powder, and chitosan/PVA nanofiber mat were analyzed.

**Figure 3:**
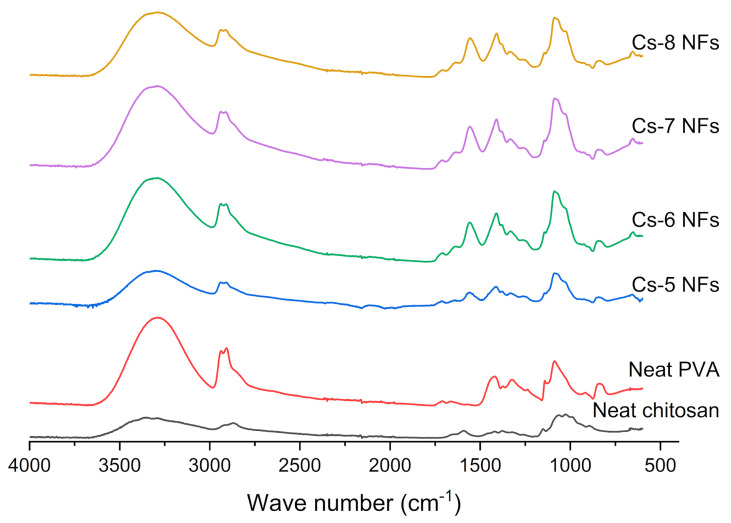
FT−IR spectrum of chitosan, PVA and chitosan/PVA nanofibers samples.

Figure 3 shows the FTIR spectra of chitosan, PVA and chitosan/PVA nanofibers (Cs-5, Cs-6, Cs-7, and Cs-8). The FTIR spectra of PVA exhibited characteristic absorption peaks at about 3290 cm^−1^ (−OH). The peak at 2937 cm^−1^ is related to antisymmetric CH_2_ stretching. Peaks at 1709 are associated with stretching vibrations of C=O bonds present in acetate units in PVA. The peak at 1420 cm^−1^ refers to the vibration of C−H of the methyl group. The peak around 1087 is related to the asymmetric stretching vibration of the C−O bond of the acetate group. The 1141 cm^−1^ is related to the −C−O group. These results are in agreement with the reported data in the literature [39]. The FTIR of chitosan shows the characteristic absorption peak at 3354 cm^−1^ assigned to O−H stretching overlapped with N−H stretching. The peak at 2926 cm^−1^ corresponds to aliphatic C−H stretching. The peak at 1561 cm^−1^ is related to the stretching vibration of the amino group. Another two characteristic peaks related to the saccharide structure of chitosan can be observed at 892 and 1150 cm^−1^; similar results have been reported in [36,40]. For all chitosan/PVA nanofibers samples, it is noticeable that the FTIR spectra are very similar to the PVA spectra, but depending on the polymer concentrations, the peaks shift and change in intensity. It was possible to observe a broad and intense peak around 3300 cm^−1^ related to O−H and N−H stretching vibrations. From the spectra, it is possible to notice the formation of a hydrogen bond between PVA and chitosan, which can be deduced by the shift toward lower values of O−H and N−H stretching vibration peak of chitosan (3354 cm^−1^) to around 3300 cm^−1^ for chitosan/PVA nanofibers. Additionally, it was possible to notice a shift from the peak from around 1590 cm^−1^ to 1560. This shifting can be related to the −NH of chitosan’s group with OH groups of PVA (1562, 1558, 1557, and 1557 cm^−1^ for Cs-5, Cs-6, Cs-7, and Cs-8, respectively), where for the samples with higher PVA concentration (Cs-5), this peak appear less intense, which can be related to hydrogen bonding between chitosan and PVA, as well. Peaks around 1709 and 1640 cm^−1^ are associated with stretching vibrations of the C=O and C−O bonds of acetate units in PVA molecules. The peak at 1420 cm^−1^ corresponds to the vibrations of the C−H bond of the methyl group (−CH_3_). The asymmetric stretching vibration of the C−O bond of the acetate group can be observed in the peak at 1085 cm^−1^. The peak around 840 cm^−1^ is associated with bending vibrations of C−H bonds in the molecule. These results are in good agreement with previous reports [36].

### 3.3. Thermal Properties

**Figure 4:**
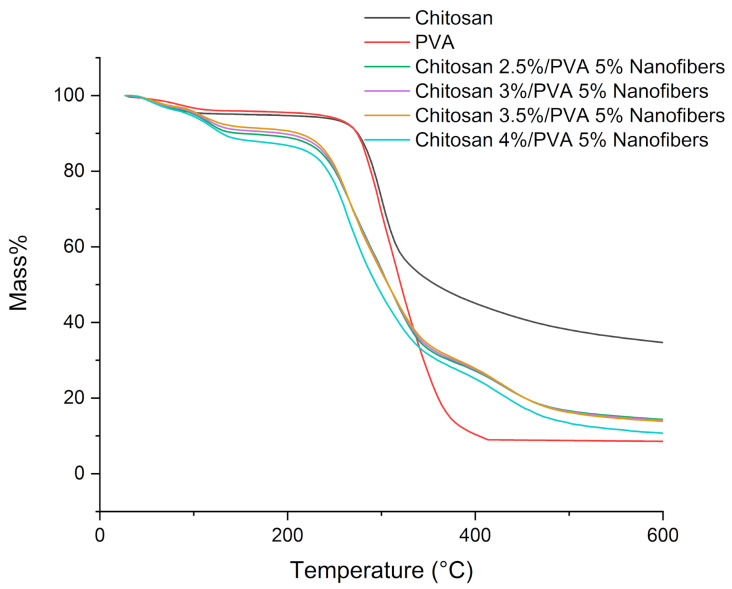
TG thermographs of chitosan, PVA, and chitosan/PVA nanofibers samples.

The TGA thermogram of chitosan/PVA nanofibers (Cs-5, Cs-6, Cs-7, and Cs-8) showed the weight loss profile at various temperatures (Figure 4). The weight loss of chitosan and PVA went through two stages. For chitosan, the first weight loss happens at 35–118 °C, corresponding to the loss of moisture (~5%), while the second weight loss took place at 182–400 °C, corresponding to thermal degradation with the deacetylation of chitosan (~49.68%) [41]. For PVA, the first weight loss occurred at 51–134 °C, related to moisture vaporization (~4%), while the second weight loss was at 157–450 °C, associated with the thermal degradation of PVA (~90.52%) [42]. TGA data for chitosan/PVA nanofibers are summarized in Table 2.

**Figure 5:**
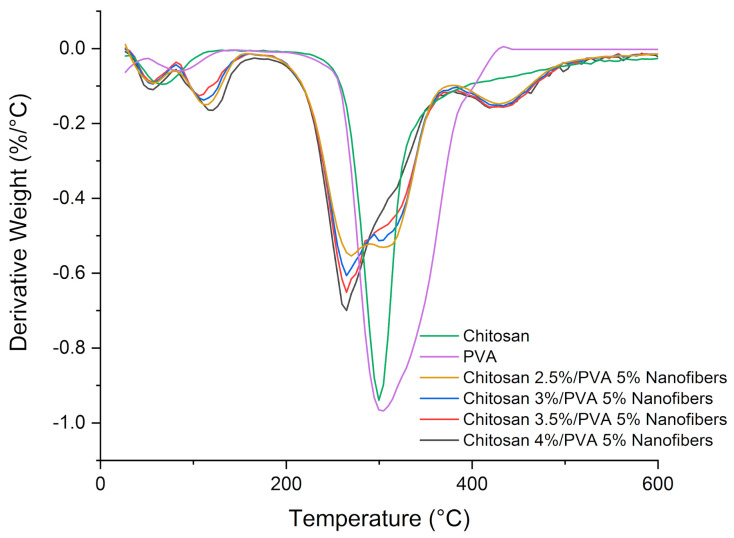
DTG thermographs of chitosan, PVA, and chitosan/PVA nanofibers samples.

The mass loss of all chitosan/PVA nanofibers samples happens in three stages, as shown in Figure 5 [43]. The first mass loss stage occurred in the range of 50–158 °C, which is related to moisture and solvent residue vaporization. From the DTG curves, it is possible to observe that two peaks are formed in this stage; this can be due to the presence of acetic acid residues, which have a higher boiling point (118 °C) in comparison to water. The second mass loss took place in the range of 180–375 °C, corresponding to the thermal degradation of chitosan and PVA. DTG curves show that the second mass loss for nanofiber samples is composed of two peaks. The first peak, observed at a temperature around ~267 °C, is related to the destruction of the chitosan/PVA complex. The second peak, observed at a temperature of ~310 °C, is related to the destruction of chitosan and PVA without any interaction. It is also noticeable that the samples with a lower chitosan content formed a lower polymeric complex between chitosan and PVA. Whereas the third mass loss observed in the range of 375–530 °C is related to the degradation of PVA byproducts generated during the thermal degradation process [44,45].

From the obtained result, it is possible to observe that the thermal stability of the electrospun nanofibers decreases as the chitosan content increases. Even more, if comparing the thermal stability of nanofibers samples with the pure PVA and chitosan, a major reduction in the thermal stability can be noticed (Table 2). The decrease in the thermal stability in the chitosan/PVA nanofibers can be explained by polymer–polymer interaction. The amorphous structure of chitosan dispersed along PVA polymeric chains generates defects in the crystalline phase of PVA, thus, hindering the development of the crystalline structure. Hence, less heat is needed to destroy the hydrogen bonding and free PVA chains to melt, which results in a lower melting point from the chitosan/PVA composites [46,47].

**Figure 6:**
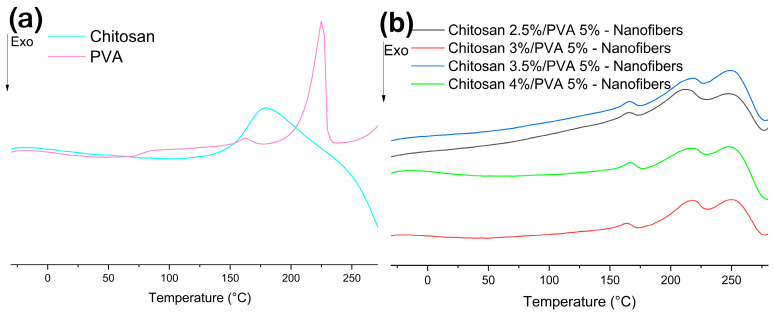
DSC thermographs of (**a**) chitosan and PVA; (**b**) chitosan/PVA nanofibers samples.

Figure 6a shows the DSC thermographs of chitosan and PVA. The DSC thermograph of chitosan shows a broad endothermic peak at 180 °C followed by an exothermic peak at 286 °C [48]. The first peak can be attributed to the molecular arrangement of chitosan chains, while the second peak corresponds to the thermal decomposition of chitosan, which is in good agreement with the data obtained from the DTG analysis, where the thermal decomposition for chitosan was found around 290 °C. From the DSC thermograph for PVA, it is possible to observe a change in the baseline at 71 °C related to glass transition [41]. The melting point peak was detected at 170 °C, within the temperature range of 150–178 °C. The characteristic peak of the crystalline polymer fraction of PVA was observed at 220 °C [49].

Figure 6b shows the DSC thermograph of chitosan/PVA nanofibers. From the DSC curves of chitosan/PVA nanofibers, it is possible to notice that the change in the baseline related to the glass transition of PVA has completely disappeared for all nanofiber samples. The orientation of the polymeric chains caused by the shear and tensile stress exerted by the electric field during the electrospinning process can be responsible for this [38]. From the DSC curves in all nanofibers samples, an endothermic peak at 165, 165, 164, and 163 °C for Cs-5, Cs-6, Cs-7, and Cs-8, respectively, can be observed. This first peak can be related to the melting point. This is followed by an exothermic peak at 231, 229, 229, and 227 °C for Cs-5, Cs-6, Cs-7, and Cs-8, respectively, which can be caused by a cross-linking (complex formation between chitosan and PVA) reaction on the chitosan molecules [36]. The third peak, an exothermic peak at 271, 268, 265, and 264 °C for Cs-5, Cs-6, Cs-7, and Cs-8, respectively, is associated with the thermal decomposition of the nanofibers, which are in good agreement with TGA results (Table 2). Although it is well known that the electrospinning process can increase the crystallinity of the electrospun polymer, this has been the case with pure PVA, as reported by Koosha et al. [36]. Nevertheless, it has been reported that the crystalline microstructures of electrospun fibers may not develop due to the high-speed solidification process of the stretched polymer. Therefore, the resulting fibers exhibit decreased crystallinity compared to their powdered and film counterparts [36,46]. In addition, another factor influencing the decrease in crystallinity of chitosan/PVA nanofibers is due to polymer–polymer interactions. As a result of hydrogen bonding between −OH groups of PVA and −NH_2_ of chitosan, the ordered structure of the PVA polymer chain is disrupted [50,51].

### 3.4. Mechanical Properties

**Figure 7:**
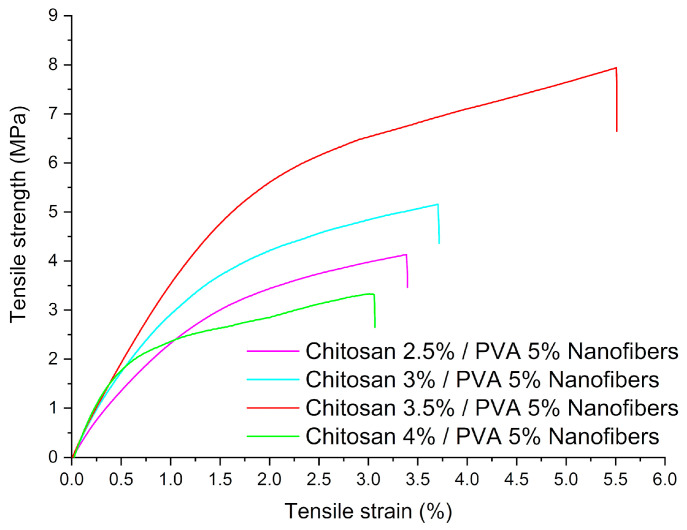
Chitosan/PVA nanofibers stress–strain diagrams.

The tensile strength, Young’s moduli, and elongation at break of chitosan/PVA nanofibrous mats are displayed in Table 3. From the obtained results, it is possible to notice that the tensile strength increased from 3.84 MPa (Cs-5) to 6.42 MPa (Cs-7) with the increase in chitosan concentration, as shown in Figure 7. It could be found that the Young’s modulus of the nanofibers samples increased from 299.7 MPa (Cs-5) to 648.45 MPa (Cs-8) with increasing chitosan concentration.

This behavior could be due to the intermolecular interaction between chitosan and PVA, where the polymer–polymer interaction is caused by hydrogen bonds between amino (−NH_2_) and hydroxyl (−OH) groups in chitosan and hydroxyl groups in PVA, as can be deduced from the FTIR data [52]. Nevertheless, it is possible to notice that tensile strength for Cs-8 decreases (2.82 MPa). This can be related to the increment of molecules with high molecular weight (~261 kDa) and the hard backbone of chitosan, which is well-known to be a rigid and brittle natural polymer [53,54]. Similar results have been reported by Bin Duan et al. [55] in their work on chitosan/PVA nanofibers electrospun from a solution with a volume ratio of 60:40 (chitosan:PVA). The tensile strength and elongation at break were 4.3 *±* 0.4 MPa and 4.3 *±* 0.6%, respectively. Similarly, Koosha M. et al. [36] reported the fabrication of PVA and chitosan/PVA nanofibers with a 30% chitosan content. Chitosan/PVA nanofibers had a tensile strength and elongation at break of 5.26 *±* 0.53 MPa and 4.5 *±* 1%, respectively. Meanwhile, PVA nanofibers had a tensile strength and elongation at break of 7.4 *±* 0.37 MPa and 50.2 *±* 5%, respectively.

### 3.5. Swelling Ratio of Chitosan/PVA Nanofiber Hydrogels

**Figure 8:**
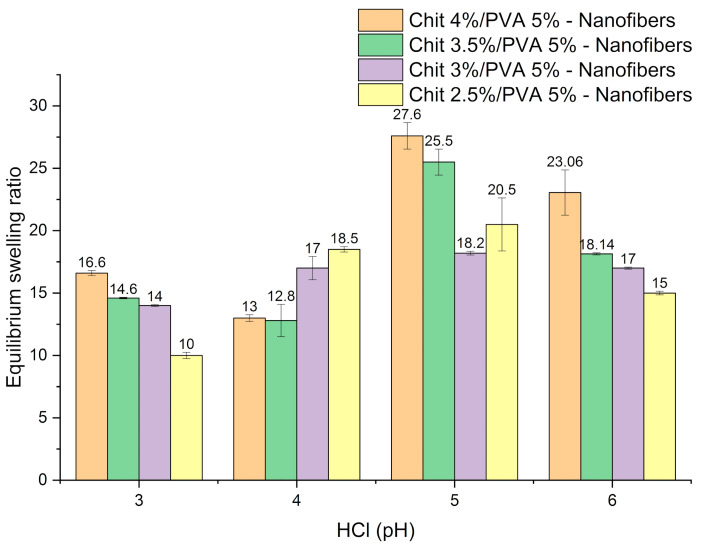
Chitosan/PVA nanofiber equilibrium swelling ratio at different HCl pH.

Figure 8 shows the swelling behavior of chitosan/PVA nanofiber hydrogels. The samples were immersed in the solution for 24 h until they swelled to equilibrium, which was reached after confirming the samples’ weight steadiness. From the obtained results, it was possible to notice that the maximum swelling for all nanofiber samples (Cs-5, Cs-6, Cs-7, and Cs-8) was obtained in the pH range 5–6. However, as the pH decreases, it is possible to notice that the swelling ratio also decreases. This behavior can be explained as follows. Amino groups in chitosan are protonated at low pH, which induces a change in chitosan structure. It has been suggested that the pKa of chitosan is in the pH range of 6–7 [56]. In this pH range, the amino groups of chitosan are ionized, and −NH_3_^+^ groups are distributed among the fiber network. Increasing the osmotic pressure between the inside and outside leads to a higher swelling degree; furthermore, the electrostatic repulsion between the chains increases the swelling degree of the nanofiber. Nonetheless, as the pH lowers, the concentration of H^+^ ions increases, leading to a reduction in the osmotic pressure, thus, decreasing the swelling behavior of the nanofibers. Moreover, the excess of Cl^-^ ions contributes to the deswelling at this pH range [57].

### 3.6. Electroactive Response of the Nanofiber Hydrogels

#### 3.6.1. Influence of Chitosan Content on the Speed Displacement at Different pH

**Figure 9:**
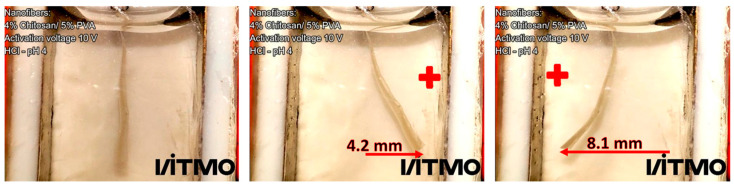
Bending deformation of chitosan/PVA nanofiber hydrogel under an applied voltage. From left to right, the electrical potential applied was equal to 0 V, 10 V, and −10 V (the electrode’s polarities were inversed), respectively.

The electroactive response of electrospun nanofiber hydrogels was studied by measuring the displacement of the samples as a function of time in an electrochemical cell. The influence of chitosan concentration and pH (from 2 to 12) on the electroactive response was analyzed. For this experiment, the samples used had a dimension of 20 × 4 mm (length × width) with a thickness of ~0.03 mm in a dry state. Figure 9 shows the bending deformation of a chitosan/PVA (Cs-8) nanofibrous hydrogel in an HCl pH 4 under an electrical potential of 10 V for 50 s, which exhibited a maximum displacement of 8.1 mm.

**Figure 10:**
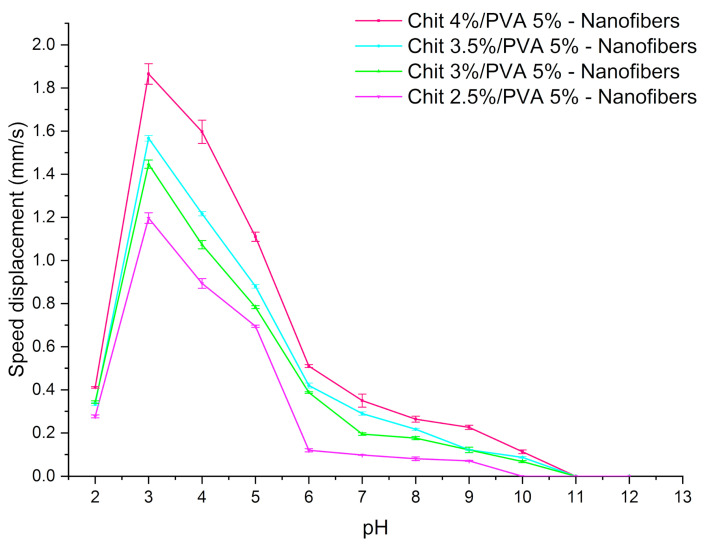
Speed displacement of nanofiber hydrogels in dependence of chitosan concentration at different pH.

It was observed that the speed displacement is strongly related to the concentrations of chitosan as well as to the pH of the electrolyte solution, as shown in Figure 10. As the chitosan concentration increased, the nanofibers became more electrical responsive, exhibiting a larger displacement at all pH solutions, reaching a maximum speed displacement of 1.86 mm s^−1^ in a pH 3. Meanwhile, the sample with the lowest chitosan concentration (2.5 wt.%) exhibited a speed displacement of 1.2 mm s^−1^ under the same conditions. This behavior can be related to the increase in free amino groups in the chitosan/PVA nanofibers complex. Furthermore, the electroactive response of the nanofibers was tested at pH > 7. Contrary to when the pH decreased from 6 to 3 and the electroactive response of the nanofibers increased, it was observed that as the pH increased from 7 to 12, the electroactive performance of the nanofibers dramatically decreased. When the solution reached a pH > 10, none of the samples showed any electroactive response (Figure 10). Briefly, the decline in the electroactive response as pH increases is related to the decrease in H^+^ available to protonate the polycationic structure of chitosan (further explanation is given in the next paragraph).

A characteristic that endows chitosan with its electroactive properties is the presence of amino groups. Amino groups within the chitosan structure are protonated in an acidic environment (pH < 7). Thus, chitosan behaves as a cationic polyelectrolyte [56]. Therefore, the bending deformation of the nanofibers under an electric field can be explained by Flory’s theory of osmotic pressure, according to the literature [57,58]. When applying an electric field, free ions are attracted to the counter electrode; this ion mobility generates a concentration gradient of mobile ions in the solution. As a result, the ionic concentration between the inside and outside of the nanofibers is different, causing the osmotic pressure of the anode side (π_1_) not to bend equally to that of the cathode side (π_2_), as a result of the osmotic pressure difference $∆\pi \left(∆\pi =\pi_{1}-\pi_{2}\right),$ the material will bend. For any polyanionic hydrogel, the bending deformation will be toward the cathode, whereas for any polycationic hydrogel, the bending deformation will be toward the anode [59,60].

#### 3.6.2. Determination of Free Amine (−NH_2_) by Spectra Deconvolution

**Figure 11:**
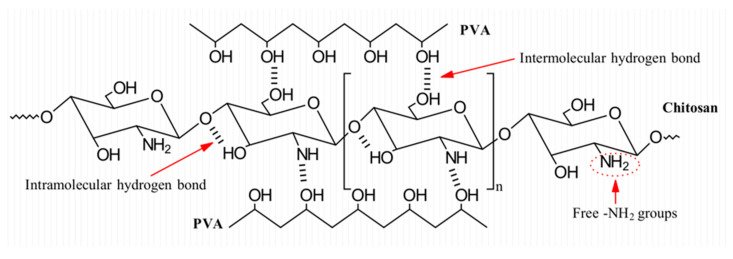
Schematic of possible intermolecular and intramolecular hydrogen bonds between chitosan and PVA.

It is known that amino groups are involved in the electroactive behavior of chitosan. As reported in many works, when chitosan is in contact with an acidic environment (pH < 7), amino groups −NH_2_ are protonated, which causes chitosan to behave as a cationic polyelectrolyte [61]. Nonetheless, when chitosan and PVA interact with one another, intramolecular and intermolecular hydrogen bonds are formed, as shown in Figure 11.

**Figure 12:**
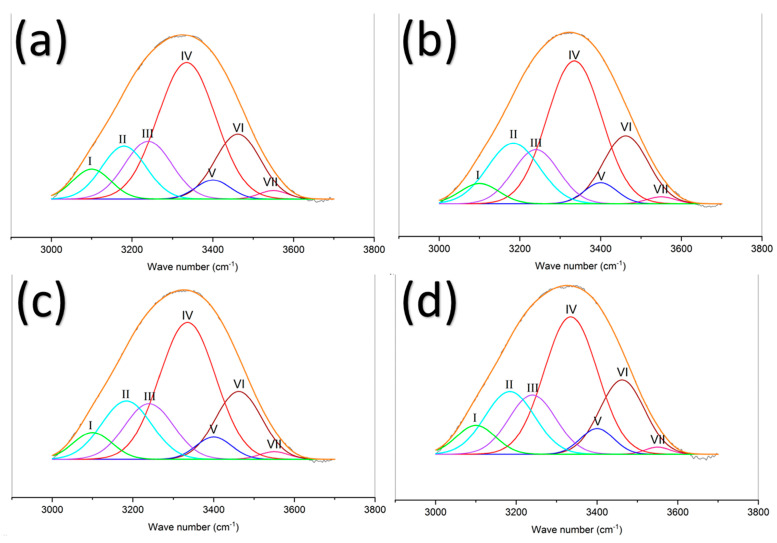
Spectra deconvolution for chitosan/PVA nanofibers samples at dry state with different chitosan content. (**a**) nanofibers with 2.5% chitosan/5% PVA; (**b**) nanofibers with 3% chitosan/5% PVA; (**c**) nanofibers with 3.5% chitosan/5% PVA; (**d**) nanofibers with 4% chitosan/5% PVA.

As a result of these interactions between polymers, the quantity of free amino groups can be reduced, hence influencing the electroactive properties of the material. Aiming to determine the hydrogen bonding interaction and the proportion of free amine, a deconvolution in Gaussian line shapes was performed on the 3000–3700 cm^−1^ peak in the FTIR spectrum (Figure 12). Hydrogen bond types were analyzed in the −OH region and the −NH region. For the −NH region, the characteristic absorption peak of the free amine group is around 3408 cm^−1^, the characteristic absorption peak of intermolecular association (N_2_−H_1_…O_5_/N_2_−H_2_…O_1_) is around 3335 cm^−1^, the characteristic absorption peak of the amide group (−CONH−) is around 3240 cm^−1^, and the characteristic absorption peak of primary ammonium (−NH^+^_3_) is around 3100 cm^−1^. For the −OH region, the characteristic absorption peak of free hydroxyl (−OH) is around 3580 cm^−1^, and the characteristic peak of multimer intermolecular association (O_6_H…N_2_) is around 3462 cm^−1^ [62,63,64,65,66]. The area under the curve is assigned to each characteristic peak which represents the composition ratio of hydrogen bonds in the −NH and −OH regions; all results are shown in Table 4.

From Table 4, it is possible to observe that the proportion of free amine increases from 3.6% to 4.59% as the concentration of chitosan increases. Additionally, it is possible to notice that the proportion of intermolecular association decreases as the chitosan content increases, reaching a minimum value of 39.15% for the nanofiber sample Cs-8 (4% chitosan/5% PVA). From these results, the electroactive behavior of the material could be better understood. As chitosan content is lower, higher hydrogen bonds are formed, these hydrogen bonding can take part in −NH_2_…−OH interactions, reducing the available free amino groups that can be protonated and be participants in the electro-activation of the chitosan/PVA nanofibers. As for the samples with a higher chitosan content, due to the larger number of amino groups, it is possible for the polymer to form −NH_2_…−OH hydrogen bonds while maintaining a greater proportion of free amino groups available to be protonated, hence increasing the electro-activation sensitivity which can be noticed in its faster speed displacement.

## 4. Conclusions

Chitosan/PVA nanofibers at different chitosan content were successfully obtained using the electrospinning method. Furthermore, the electrospun nanofibers demonstrated the potential to be used as electroactive nanofibrous actuators. The developed nanofiber actuators showed a fast tip displacement under a low voltage, reaching a maximum speed displacement of 1.86 mm s^−1^ at a pH 3 and a voltage of 10 V. The fact that nanofibers have a higher response may be related to their one-dimensional morphology. This increases the exposure of −NH_2_ groups to the acidic environment. Consequently, the protonation of amino groups in the material is favored. The results on the electroactive response were supported by deconvoluting the FTIR spectra in the 3000–3700 cm^−1^ region. Deconvolution showed that the proportion of free amine changes in relation to the chitosan; samples with lower chitosan content had a lower proportion of free amine (3.6%), whereas nanofiber samples with higher chitosan content had a higher proportion of free amine (4.59%). From the electroactive response experiments, it was possible to observe a dependence on the chitosan content and the pH with the bending activation of the nanofibers. This study demonstrated that chitosan/PVA nanofiber is a potential material for the development of soft actuators using biopolymers as base material, which can be useful not only in the field of soft robotics but, due to chitosan’s biocompatibility, can open the possibility for its application in the biomedical field. Some of its potential applications can be found in the development of soft grippers for the manipulation of small objects immersed in an aqueous environment, the fabrication of electroresponsive scaffolds, or the development of microrobotic systems.

## Figures and Tables

**Table 1:** Morphology and diameter distribution of chitosan/PVA fibers.

Sample	Chitosan (wt.%)	PVA (wt.%)	Mean (µm)	Morphology
Cs-5	2.5	5	0.617 ± 0.1	High presence of particles and fibers
Cs-6	3	5	0.539 ± 0.09	Presence of beads, with a high formation of uniform fibers
Cs-7	3.5	5	0.523 ± 0.09	High presence of beads, with a uniform fiber formation
Cs-8	4	5	0.581 ± 0.1	Uniform fiber formation

**Table 2:** Thermogravimetric analysis of chitosan/PVA fibers.

Sample	Chitosan (wt.%)	PVA (wt.%)	First Mass Loss (%) to 50–158	Second Mass Loss (%) to 180–375	Third Mass Loss (%) to 375–530	First Stage 1st Peak (°C)	First Stage 2nd Peak (°C)	Second Stage 1st Peak (°C)	Second Stage 2nd Peak (°C)	Third Stage (°C)
PVA	-	-	4	90.53	-	81	-	304	-	-
Chit	-	-	5	49.68	-	67	-	299	-	-
Cs-5	2.5	5	10.2	59.18	13.06	56.6	113.4	270.5	310	431
Cs-6	3	5	8.7	59.66	14	56	111.21	267.0	310	430
Cs-7	3.5	5	8	60.4	14.98	56.3	106.6	265.8	309	428
Cs-8	4	5	11.1	59.1	15	55.95	120.5	263.9	-	428

**Table 3:** Tensile strength and Young’s modulus of chitosan/PVA nanofibers samples.

Sample	Chitosan (wt.%)	PVA (wt.%)	Young’s Modulus (MPa)	Tensile Strength (MPa)	Elongation at Break (%)
Cs-5	2.5	5	299.7 ± 7.65	3.84 ± 0.39	3.37 ± 0.29
Cs-6	3	5	402.8 ± 4.07	4.27 ± 0.59	3.24 ± 0.46
Cs-7	3.5	5	439.35 ± 10.45	6.42 ± 0.8	5.92 ± 0.69
Cs-8	4	5	648.45 ± 12.04	2.82 ± 0.27	3.12 ± 0.42

**Table 4:** List of peak frequencies and relative strength of the deconvoluted band in the region 3000–3700 cm^−1^ for chitosan/PVA nanofibers samples with different chitosan content at dry state.

Sample	Hydrogen Bond Types		Abbreviation	Wavenumber/cm^−1^	Relative Strength/%
Cs-5	Primary ammonium	I	−NH^+^_3_	~3100 cm^−1^	6.31
	Intermolecular hydrogen bond	II	OH…ether O	~3200 cm^−1^	13.14
	Amide	III	−CONH−	~3240 cm^−1^	15.11
	Intermolecular association	IV	N_2_−H_1_…O_5_/N_2_−H_2_…O_1_	~3335 cm^−1^	43.90
	Free amine	V	−NH_2_	~3408 cm^−1^	3.66
	Multimer (Intermolecular association)	VI	O_6_H…N_2_*	~3462 cm^−1^	16.48
	Free hydroxyl	VII	−OH	~3580 cm^−1^	1.36
Cs-6	Primary ammonium	I	−NH^+^_3_	~3100 cm^−1^	4.23
	Intermolecular hydrogen bond	II	OH…ether O	~3200 cm^−1^	17.30
	Amide	III	−CONH−	~3240 cm^−1^	13.64
	Intermolecular association	IV	N_2_−H_1_…O_5_/N_2_−H_2_…O_1_	~3335 cm^−1^	42.91
	Free amine	V	−NH_2_	~3408 cm^−1^	3.79
	Multimer (Intermolecular association)	VI	O_6_H…N_2_*	~3462 cm^−1^	17.01
	Free hydroxyl	VII	−OH	~3580 cm^−1^	1.08
Cs-7	Primary ammonium	I	−NH^+^_3_	~3100 cm^−1^	5.54
	Intermolecular hydrogen bond	II	OH…ether O	~3200 cm^−1^	15.72
	Amide	III	−CONH−	~3240 cm^−1^	14.54
	Intermolecular association	IV	N_2_−H_1_…O_5_/N_2_−H_2_…O_1_	~3335 cm^−1^	41.62
	Free amine	V	−NH_2_	~3408 cm^−1^	4.29
	Multimer (Intermolecular association)	VI	O_6_H…N_2_*	~3462 cm^−1^	16.95
	Free hydroxyl	VII	−OH	~3580 cm^−1^	1.21
Cs-8	Primary ammonium	I	−NH^+^_3_	~3100 cm^−1^	5.94
	Intermolecular hydrogen bond	II	OH…ether O	~3200 cm^−1^	16.67
	Amide	III	−CONH−	~3240 cm^−1^	14.85
	Intermolecular association	IV	N_2_−H_1_…O_5_/N_2_−H_2_…O_1_	~3335 cm^−1^	39.15
	Free amine	V	−NH_2_	~3408 cm^−1^	4.59
	Multimer (Intermolecular association)	VI	O_6_H…N_2_*	~3462 cm^−1^	17.82
	Free hydroxyl	VII	−OH	~3580 cm^−1^	0.95

## Data Availability

The data presented in this study are available on request from the corresponding author.
